# Assessment of Different Types of Strabismus Among Pediatric Patients in a Tertiary Hospital in Jeddah

**DOI:** 10.7759/cureus.11978

**Published:** 2020-12-08

**Authors:** Ahmed S Qanat, Abdullah Alsuheili, Abdulkarim M Alzahrani, Abdulrahman A Faydhi, Abdulhadi Albadri, Nizar Alhibshi

**Affiliations:** 1 Research, King Abdulaziz University Hospital, Jeddah, SAU; 2 Ophthalmology, King Abdulaziz University Hospital, Jeddah, SAU

**Keywords:** strabismus, pediatric, esotropia, exotropia, jeddah, saudi arabia, hypertropia, dissociated vertical deviation

## Abstract

Background

Strabismus, also known as squint, is an ocular disorder in which the eyes do not align properly with each other when looking at an object. The estimated global prevalence of strabismus among children is between 1.3% and 5.7%. This study aimed to assess the various types of strabismus among pediatric patients in Jeddah, in the western region of Saudi Arabia.

Methods

The medical records of 281 patients with strabismus aged ≤18 years, who presented to the pediatric ophthalmology clinic in King Abdulaziz University Hospital between 2010 and 2019, were retrospectively reviewed. Data were analyzed using the Statistical Package for Social Sciences (SPSS; IBM Corp., Armonk, NY, USA). A p-value of 0.05 or less was considered statistically significant.

Results

Out of the 281 patients, 141 were (50.2%) female. The average age of the patients was 9.50 ± 4.24 years. The most common type of strabismus was esotropia (177 [63%] patients), followed by exotropia (96 [34.2%] patients), hypertropia (10 [3.6%] patients), and dissociated vertical deviation (four [1.4%] patients). Two-hundred thirty-one (82.8%) patients had bilateral strabismus. A total of 178 patients (63.3%) had no associated conditions with strabismus, whereas 103 (36.7%) had an associated condition. A significant relationship was observed between esotropia and prematurity (p = 0.024).

Conclusion

Esotropia was the most common type of strabismus among the patients, followed by exotropia. The results of this study showed that males were equally affected as females. We also found a significant relationship between esotropia and prematurity. Implementation of a compulsory nationwide pediatric ophthalmic screening program for children aged one, three, and five years is recommended to enable timely diagnosis of strabismus and any other refractive errors.

## Introduction

Strabismus, commonly referred to as squint, cross-eyes, walleyes, wandering eyes, or deviating eyes [1], is an ocular condition characterized by misalignment of the eyes when viewing an object; this misalignment may be continuous or intermittent [2]. Based on the direction of the deviated eye, strabismus may be classified as esotropia (inward deviation), exotropia (outward deviation), hypertropia (upward deviation), or hypotropia (downward deviation) [3]. An infant with strabismus may stop using the impaired eye, resulting in a visual impairment termed as amblyopia, which may become permanent if not treated early. Since strabismus may also inhibit the development of binocular vision, early intervention is vital to ensure optimal visual function [1,4]. Due to the visual problems associated with it, strabismus may leave a negative psychosocial impact on the child. In addition, children with strabismus have a reduced vision-related quality of life in comparison to children with normal vision. Moreover, the parents of these children also experience a reduced quality of life [5,6]. Strabismus is associated with various risk factors such as low gestational age, reduced birth weight, family history of squint [7], neuromuscular disorders, maternal smoking during pregnancy, cataracts, head injuries, tumors of the brain or eyes, and systemic conditions that have ocular manifestations [1].

Strabismus is not a rare condition, and the global prevalence among children is about 1.3%-5.7% [8]. In a study conducted in Saudi Arabia in 2015 to assess the pattern of eye diseases among pediatric patients aged one to 14 years, the estimated prevalence of strabismus was reported as 11.8% [9]. In a retrospective, population-based, cohort study conducted in Olmsted County, Minnesota, USA, 627 new cases of childhood squint were recognized during the 10-year study period, which included 380 (60.1%) cases of esotropia, 205 (32.7%) of exotropia, and 42 (6.7%) of hypertropia [10]. In contrast, a Japanese study has shown different prevalence statistics, in which exotropia was predominant [11]. Additionally, a population-based study conducted in Palestine, Gaza Strip, in 2014, indicated esotropia as the most prevalent type of squint [12].

Regarding studies conducted in Saudi Arabia, a study on 385 patients who presented to the pediatric ophthalmology clinic in Jazan, Saudi Arabia, between October 2014 and October 2015 revealed that strabismus (36.9%) was the most common eye disorder observed [13]. A recent cross-sectional study conducted on 156 randomly selected participants in Arar, northern Saudi Arabia, showed that 14.7% of them were strabismic; among these, 78.3% had esotropia, and 17.4% had exotropia. The study further demonstrated that strabismus has a significant relationship with consanguinity [14]. In a study conducted in the middle region of Saudi Arabia, which included 4,886 patients with strabismus who had undergone surgery in Riyadh, esotropia was the most common type of strabismus (69.3%), whereas exotropia was less prevalent (26.9%) [15]. A clinic-based study performed in the eastern region of Saudi Arabia in 2015, which included 1,350 children, revealed that 38% of the participants had strabismus [16].

A search of the available literature revealed limited local data regarding the prevalence of strabismus among children in Jeddah, which is in the western region of Saudi Arabia. To fill this gap, we conducted this study to assess the various types of strabismus among pediatric patients in Jeddah.

## Materials and methods

This was a retrospective medical record review study. The study was approved by the institutional review board of King Abdulaziz University Hospital (KAUH) (Reference Number 304-20). No names or identification numbers were recorded to ensure patient confidentiality. All patients provided informed consent to participate in this study. We recruited patients aged 18 years or less who were seen at the ophthalmology clinic of our hospital and were diagnosed with strabismus. This study was conducted between June and September 2020. 

A total of 471 medical records from 2010 to 2019 were reviewed; among these, 281 patients met our inclusion criteria. Patients with missing data were excluded from the study. Data were extracted from the electronic-based medical record system of KAUH (Phoenix). The extracted data included demographic data, age at the time of diagnosis, type of strabismus, test/s performed, laterality of the affected eye, and other associated conditions. The patients were categorized into three groups according to their age at the time of this study: one to six years, seven to 12 years, and 13 to 18 years. 

All data were entered into Microsoft Excel version 2010. The Statistical Package for Social Sciences (SPSS) version 21 (IBM Corp., Armonk, NY, USA) was used for data analysis. Categorical variables were expressed as frequencies and percentages, whereas continuous data were presented as means ± standard deviations. The chi-square test was used to calculate the correlations between variables. A p-value of 0.05 or less was considered statistically significant.

## Results

A total of 281 medical records of pediatric patients with strabismus were reviewed; 141 (50.2%) patients were female and 140 (49.8%) were male. Out of the 281 patients, 207 (73.7%) were Saudi nationals, whereas 74 (26.3%) were not. The mean age of the patients was 9.50 ± 4.24 years and the mean age at the time of their diagnosis was 5.19 ± 3.53 years. The seven to 12 years age group had the highest frequency with 140 (49.8%) patients, followed by the one to six years group with 75 (26.7%) and the 13 to 18 group with 66 (23.5%) patients. The most common type of strabismus was esotropia (177 [63%] patients), followed by exotropia (96 [34.2%] patients) (Table 1).

**Table 1:** Type of squint and demographics of the sample (n=281).

Variables		Type of strabismus			
		Esotropia No. (%)	Exotropia No. (%)	Hypertropia No. (%)	Dissociated vertical deviation No. (%)
Age groups	1-6 years	56 (31.6)	16 (16.7)	3 (30)	1 (25)
	7-12 years	91 (51.4)	47 (49)	3 (30)	1 (25)
	13-18 years	30 (16.9)	33 (34.4)	4 (40)	2 (50)
Gender	Male	89 (50.3)	48 (50)	5 (50)	3 (75)
	Female	88 (49.7)	48 (50)	5 (50)	1 (25)
Nationality	Saudi	135 (76.3)	64 (66.7)	10 (100)	2 (50)
	Non-Saudi	42 (23.7)	32 (33.3)	0 (0)	2 (50)
Affected eye	Unilateral	23 (13.1)	17 (17.9)	8 (80)	0 (0)
	Bilateral	153 (86.9)	78 (82.1)	2 (20)	4 (100)
Total (%)		177 (63)	96 (34.2)	10 (3.6)	4 (1.4)

Among the 281 patients, 231 (82.8%) showed bilateral involvement, whereas 48 (17.1%) showed unilateral involvement. The most common ophthalmic tests employed in our study were the cover test, followed by cycloplegic refraction, Hirschberg test, and Krimsky test, which were performed in 92 (32.7%), 63 (22.4%), 44 (15.7%), and 27 (9.6%) patients, respectively. Table 2 shows that 178 (63.3%) patients had no associated conditions with strabismus, whereas 103 (36.7%) had associated conditions. The most common condition associated with squint was developmental delay.

**Table 2:** Frequency and percentage of associated conditions. ADHD: Attention deficit hyperactivity disorder; ROOHAD: rapid-onset obesity with hypothalamic dysregulation, hypoventilation, and autonomic dysregulation.

Associated conditions		Frequency	Percentage %
Psychiatric/developmental disorders:	Developmental delay	29	10.3
	Autism	2	0.7
	ADHD	2	0.7
	Mental retardation	1	0.4
Neurological disorders:	Epilepsy	16	5.7
	Hydrocephalus	16	5.7
	Cerebral palsy	12	4.3
	Brain tumor	4	1.4
Ophthalmic disorders:	Cataract	6	2.1
	Glaucoma	3	1.1
	Optic atrophy	2	0.7
	Duane syndrome	1	0.4
Infectious disorders:	Hepatitis C	7	2.5
	Congenital toxoplasmosis	1	0.4
	Infectious mononucleosis	1	0.4
Endocrine disorders:	Hypothyroidism	14	5
	Diabetes Mellitus	3	1.1
Syndromes:	Down syndrome	7	2.5
	Alagille syndrome	1	0.4
	Dandy-walker syndrome	1	0.4
	William’s syndrome	1	0.4
	Kabuki syndrome	1	0.4
	ROOHAD syndrome	1	0.4
	Freeman-Sheldon syndrome	1	0.4
Other:	Prematurity	23	8.2
	Craniofacial anomalies	11	3.9
	Anemia	9	3.2
	Asthma	3	1.1
	Low birth weight	1	0.4
	Chiari malformation	1	0.4
Non		178	63.3

No significant relationship was observed between the affected eye (unilateral or bilateral) and exotropia or dissociated vertical deviation; however, a significant association was observed with esotropia and hypertropia (p = 0.026 and p = 0.000, respectively). No significant relationship was observed between developmental delay and esotropia or exotropia (p = 0.925 and p = 1.00, respectively). However, there was a significant association between prematurity and esotropia and exotropia (p = 0.024 and p = 0.037, respectively).

Esotropia was the most common condition in the one to six (56 [74.7%] patients) and sven to 12 (91 [65%] patients) age groups. Exotropia was most common in the 13 to 18 years age group (33 [50%] patients). We observed a significant relationship between esotropia, exotropia, and all age groups (p = 0.001 for esotropia; p = 0.002 for exotropia). No significant association was observed between sex and any type of strabismus.

## Discussion

In this study, we assessed the various types of strabismus among pediatric patients in Jeddah; esotropia was observed to be the most predominant type of strabismus among these patients. This finding is in line with those of other studies conducted in Arar city and Riyadh, Saudi Arabia [14,15]. Similar results were reported in a Palestinian study [12], the study by Medghalchi [17], Mvogo et al. [18], and the study conducted in Olmsted County, USA [10]. In contrast, exotropia is reportedly more common than esotropia in East Asia. Two Chinese studies reported exotropia to be the most common type of strabismus, with a percentage of 80.94% reported in one study and 94.4% in the other [19,20]. Additionally, exotropia is reportedly the most common type of strabismus in Japan [11]. Furthermore, other studies conducted in Iran have indicated that exotropia is the most common type of strabismus in the country [21,22].

In contrast to the study conducted in Arar, in which bilateral involvement of the eyes was not common, most of the patients in our study had bilateral strabismus [14]. This disparity may be attributed to the differences in the study designs. The Arar study was a cross-sectional population-based study; the questionnaires used in the study were completed by either the mothers of the children or the adolescents themselves. We believe that this may have made the study vulnerable to recall bias, as the participants (or their mothers) may not have noticed that the squint was bilateral. In our study, we retrieved the data, which was documented by an ophthalmologist after ophthalmic examination, from the hospital’s electronic record system.

Furthermore, we did not observe any significant association between sex and any type of strabismus in our study. A global meta-analysis, in which 56 articles were reviewed, has also shown no association between sex and the prevalence of squint [23]. We believe that this lack of association between sex and strabismus in previous studies has been a consequence of the approximate prevalence of strabismus between males and females.

The results of our study showed an association between prematurity and esotropia, which is supported by that of a study conducted in Germany [7], and that of a study by Gursoy et al. [24]. A study conducted in Baltimore, Maryland, USA, has suggested that prematurity is independently related to elevated risk for both esotropia and exotropia. However, with regard to strabismus and prematurity, it may be difficult to compare the findings of our study with those of previous studies due to the differences in sampling methods, diagnostic methods, and definitions of prematurity and squint [25]. Moreover, a prospective study has shown that the mechanism behind the relationship between prematurity and strabismus is unclear [26]. However, another study has suggested that retinopathy of prematurity during early gestational age is a predisposing factor that may lead to strabismus [27].

Although developmental delay was the most common condition associated with strabismus in our study, the association was not statistically significant. This finding was in contrast with that of previous studies [28,29], which have indicated that strabismus is a common disorder significantly associated with developmental delay in children. This variation in the results may have been due to selection bias, the differences in sampling methods, and the different study settings. KAUH is a government tertiary hospital; therefore, most of the patients are from different ethnic groups with varied socio-economic backgrounds.

Our study revealed a significant relationship between esotropia, exotropia, and all pediatric age groups. This finding differs from that of a study conducted in Jazan, Saudi Arabia, in which the 0 to six years age group had the largest number of strabismus patients [13]. Moreover, according to a retrospective study performed in Sweden, the greatest prevalence of strabismus was observed in children aged four years old, followed by a declining trend. This was achieved through an ophthalmic screening program that begins at the age of four [30]. 

However, due to the lack of visual screening programs, different ethnic backgrounds of the residents, and the shortage of research on populations within the same age groups in Saudi Arabia, the findings of the abovementioned study may be complicated and difficult to elaborate in the current study. 

Compared to the majority of previous studies, which studied strabismus in all age groups, the findings of our study add to a growing body of literature on the most common type of strabismus among the pediatric population. However, there are some limitations to this study. First, there were some difficulties in finding articles on similar topics and with similar methodologies in the available literature. Second, the documentation of visual acuity and family history in the hospital system was not sufficiently detailed. Lastly, there is a possibility of selection bias in the study due to its retrospective nature.

## Conclusions

In this retrospective study, no significant association was observed between sex and prevalence of strabismus. Further, esotropia was the most common type of strabismus, followed by exotropia. Moreover, a significant association was observed between esotropia and prematurity.

Early screening and detection of strabismus is crucial for the proper management of strabismus and strabismic amblyopia. We recommend that a compulsory nationwide pediatric ophthalmic screening program for children aged one, three, and five years be carried out to enable timely diagnosis of strabismus and other refractive errors. Parent education may be promoted through the distribution of pamphlets on amblyopia and strabismus in clinics, hospitals, and medical centers. Periodic educational programs on pediatric visual problems and the importance of early detection and management, including screening when possible, should be conducted at different locations, including schools, shopping malls, and social networks. Cycloplegic refraction should be mandatory during early childhood for patients with strabismus to assess their total amount of actual refractive errors and to help clinicians determine the best treatment method for strabismus. Further studies are needed to clarify and understand the heritability of strabismus.
